# Purinergic Signaling in the Vertebrate Olfactory System

**DOI:** 10.3389/fncel.2019.00112

**Published:** 2019-04-16

**Authors:** Natalie Rotermund, Kristina Schulz, Daniela Hirnet, Christian Lohr

**Affiliations:** Division of Neurophysiology, University of Hamburg, Hamburg, Germany

**Keywords:** purinergic signaling, olfactory system, olfaction, ATP, adenosine receptor, P2X receptor, P2Y receptor

## Abstract

Adenosine 5′-triphosphate (ATP) is an ubiquitous co-transmitter in the vertebrate brain. ATP itself, as well as its breakdown products ADP and adenosine are involved in synaptic transmission and plasticity, neuron-glia communication and neural development. Although purinoceptors have been demonstrated in the vertebrate olfactory system by means of histological techniques for many years, detailed insights into physiological properties and functional significance of purinergic signaling in olfaction have been published only recently. We review the current literature on purinergic neuromodulation, neuron-glia interactions and neurogenesis in the vertebrate olfactory system.

## Introduction

Since the discovery of adenosine 5′-triphosphate (ATP) release from sensory nerve terminals (Holton, 1959) and peripheral purinergic neurotransmission (Burnstock et al., 1970), ATP has been described as a (co-)transmitter in most if not all parts of the vertebrate nervous system. ATP is stored in and released from synaptic vesicles but alternative release pathways such as diffusion through connexin and pannexin hemichannels, volume-regulated anion channels and P2X_7_ receptor channels have been published (Bodin and Burnstock, 2001; Taruno, 2018). Once released into the extracellular space, ATP activates a specific subclass of purinoceptors, the P2 receptors. P2 receptors can further be subdivided into ionotropic P2X receptors and metabotropic P2Y receptors (Burnstock et al., 2011). P2X receptors comprise seven subtypes, named P2X_1_ to P2X_7_, that form non-selective cation channels with high calcium permeability (North, 2016; Schmid and Evans, 2019). In the extracellular space, ATP immediately undergoes enzymatic degradation. Remarkably, a variety of purinoceptors is additionally, if not exclusively, sensitive for the degradation products. The first step of enzymatic ATP degradation yields adenosine 5′-diphosphate (ADP), which is able to activate P2Y receptors in addition to ATP. Some P2Y receptors are also activated by the pyrimidines uridine 5′-triphosphate (UTP) and uridine 5′-diphosphate (UDP; Abbracchio et al., 2006). At least nine different P2Y receptors are expressed in vertebrates, however, P2Y_3_ appears to be an avian-specific receptor (Webb et al., 1996). P2Y_1,2,4,6,11,12,13,14_ are found in mammals and many of them are expressed by brain cells. The primary intracellular targets of P2Y receptors are heterotrimeric G proteins, in particular, those containing Gα_q/11_ (P2Y_1,2,4,6,11_) and Gα_i_ (P2Y_12,13,14_; Abbracchio et al., 2006; Köles et al., 2011). Hence, activation of P2Y receptors results in PLC-mediated calcium signaling and PKC activation as well as inhibition of adenylate cyclase. Besides P2 receptors, the family of purinoceptors comprises P1 receptors (Burnstock et al., 2011). P1 receptors are activated by adenosine, which is usually derived from extracellular degradation of ATP, ADP and AMP by ecto-nucleotidases (CD39/NTPDases, E-NPP, CD73) and alkaline phosphatase (Abbracchio et al., 2009; Zimmermann et al., 2012). P1 are subdivided into A_1_, A_2A_, A_2B_ and A_3_ receptors. Mostly, A_1_ and A_3_ are linked to G_i_ proteins, while A_2A_ and A_2B_ are linked to G_s_ proteins exerting an impact on the cellular cAMP levels. To finally terminate the purinergic signaling cascade, adenosine is removed by adenosine transporters or converted by adenosine deaminase and adenosine kinase to inosine and AMP, respectively (Boison, 2013; Bagatini et al., 2018; Pastor-Anglada and Pérez-Torras, 2018). Thus, in contrast to other neurotransmitter systems, the degradation of the neurotransmitter ATP does not immediately terminate transmission but generates the neuromodulators ADP and adenosine, which possess their own subsets of receptors and evoke additional cellular responses. Hence, purinergic signaling is by far more complex than other neurotransmitter systems and is determined by the combination of purinoceptors, ATP/adenosine-degrading enzymes and nucleotide/nucleoside transporters. Since this combination changes during development and circadian rhythm and differs significantly between different brain regions and even between microenvironments, purinergic signaling has multiple facets and fulfills a plethora of functions. Postsynaptic P2X receptors, e.g., mediate fast synaptic transmission (Edwards et al., 1992; Evans et al., 1992; Pankratov et al., 2007), whereas P2Y and P1 receptors rather act as neuromodulators. Adenosine, for instance, is a well-known modulator of presynaptic neurotransmitter release at central synapses (Snyder, 1985; Sebastião and Ribeiro, 2009, 2015). In addition, P2Y receptors inhibit presynaptic calcium channels, thereby reducing neurotransmitter release (Sperlágh et al., 2007; Guzman and Gerevich, 2016). Both P1 and P2 receptors are not only expressed by neurons, but also by glial cells (Deitmer et al., 1998; Verkhratsky et al., 2009; Boison et al., 2010). Activation of purinoceptors induces calcium signaling in astrocytes, which triggers the release of gliotransmitters and mediates neurovascular coupling (Pelligrino et al., 2011; Lohr et al., 2014; Köles et al., 2016). Purinergic signaling is also involved in sensory systems, both in sensory organs such as the eye and the cochlea as well as in brain regions contributing to sensory information processing (Housley et al., 2009; Lohr et al., 2011, 2014). In this review article, we summarize recent data on the role of purinergic signaling in cell physiology, adult neurogenesis and odor information processing in the vertebrate olfactory system. Purinergic signaling has also been shown to play important roles in the development of the nervous system (Zimmermann, 2011). However, studies in the olfactory system, even when performed in the developing animal, rarely address the functional role of purines in development, for which reason we will not elaborate on this topic.

## Structure of the Vertebrate Olfactory System

### Olfactory Epithelium

**Figure 1:**
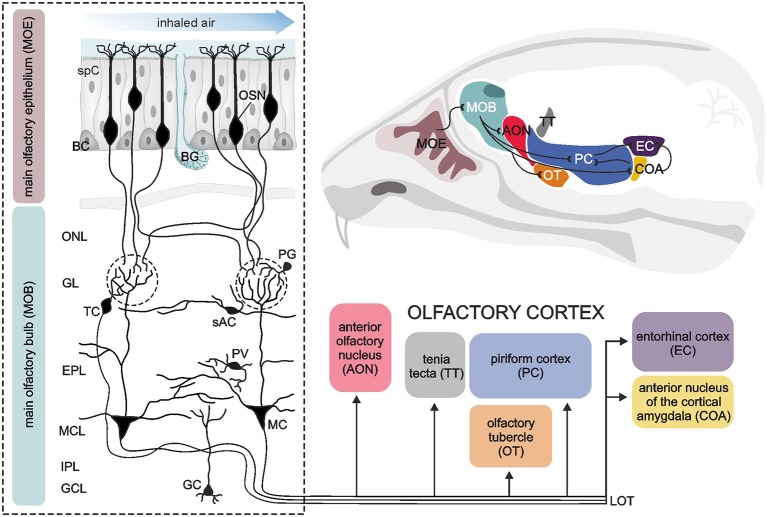
Sensory pathways in the vertebrate olfactory system. A sagittal section of the murine brain (upper right corner) illustrates the components of the vertebrate olfactory system and the connections amongst them (black lines). The dashed box on the left shows a detailed view of the neuronal architecture of the main olfactory epithelium (MOE) and the main olfactory bulb (MOB). The MOE mainly consists of olfactory sensory neurons (OSN), supporting cells (spC) and basal cells (BC) as well as Bowman‘s glands (BG) providing a mucosa covering the MOE. Volatile odorant molecules are drawn to the nasal cavity by sniffing, pass the mucosa and are detected by OSN. OSN project their axons to the MOB. After passing the olfactory nerve layer (ONL), all OSN expressing the same olfactory receptor converge to the same glomerulus (dashed circles), where the information is synaptically transmitted to second-order neurons, mitral (MC) and tufted cells (TC). The MOB also harbors different types of interneurons, such as periglomerular neurons (PG), short axon cells (sAC), parvalbumin-positive neurons (PV) and granule cells (GC). These interneurons form complex synaptic networks with MC and TC, particularly in the glomerular layer (GL) and the adjacent external plexiform layer (EPL). The somata of MC are located in the mitral cell layer (MCL). The internal plexiform layer (IPL) mainly consists of axons from MC and TC, forming the lateral olfactory tract (LOT). MC and TC generate output *via* LOT and project their axons to different brain areas referred to as the olfactory cortex, consisting of the anterior olfactory nucleus, tenia tecta, olfactory tubercle, piriform cortex, anterior nucleus of the cortical amygdala and the entorhinal cortex. Illustration by SciGraphics.

The vertebrate main olfactory system can be divided into distinct hierarchical levels, mainly the olfactory epithelium, the olfactory bulb and the olfactory cortex (Figure 1). In addition, the vomeronasal organ is also part of the vertebrate olfactory system (forming the sensory organ of the additional olfactory system) conveying the detection of pheromones, but we will focus here on the main olfactory system. Odor sensation is initiated by odor molecules flooding the nasal cavity and their detection by olfactory sensory neurons embedded in the olfactory epithelium, a specialized epithelial tissue located in the nasal cavity. Four main cell types provide its functionality; sensory neurons, basal cells, sustentacular cells and microvillar (brush) cells. Olfactory sensory neurons are bipolar neurons with apical poles equipped with cilia that reach into the mucosa covering the epithelium. On the cilia, odorant receptors are located which are activated by odor molecules resulting in excitation of olfactory sensory neurons. Specialized odorant-binding proteins facilitate the solubility of odor molecules in the mucus and therefore are actively involved in the availability of odor molecules for the odorant receptors (Heydel et al., 2013; Pelosi et al., 2014). Since the sensory neurons of the olfactory epithelium are in constant contact with respiratory air containing pathogens and hazardous substances, they have a very limited span of life and have to be replaced by newborn neurons generated from basal cells every few weeks (Brann and Firestein, 2014). Basal cells are stem cells located nearby the basal lamina of the olfactory epithelium. Microvillar cells are microvilli-bearing columnar cells that contact the afferent nerve endings of the trigeminal nerve with their basal surface and mediate the transduction of general sensation (Lucero, 2013). In addition to these cells, the undermost layer of the olfactory epithelium, the lamina propria, harbors olfactory glands (Bowman’s glands). They deliver the protein-rich secret onto the air-facing side of the epithelium. Supporting cells, as the name suggests, are non-neuronal cells providing metabolic and physical support for the cell types described above. The axons of olfactory sensory neurons project into the central nervous system forming the first cranial nerve. Many small nerve fascicles pass the perforated cribriform plate to enter the cranial cavity and eventually spread over the surface of their destination area, the olfactory bulb.

### Olfactory Bulb

The vertebrate olfactory bulb, the first relay station of the olfactory sensory pathway, is a highly structured area of the forebrain with a complex cellular architecture. Morphologically and functionally distinct layers harbor diverse specialized populations of neurons and glial cells (Figure 1). The most superficial layer of the olfactory bulb is the olfactory nerve layer. It is composed of sensory axons, which are assembled into fascicles and surrounded by a unique type of glial cells, the olfactory ensheathing cells. Eventually, the axons of the olfactory sensory neurons exit the nerve layer towards deeper layers to terminate in the glomerular layer. Here, they synapse onto apical dendrites of mitral and tufted cells in so-called glomeruli, separated globular neuropil compartments that form distinct processing units for the incoming odor signals. Mitral and tufted cells, the projection neurons of the olfactory bulb, are often regarded as functionally homogenous and therefore collectively named mitral/tufted cells. In addition to sensory axons and mitral/tufted cell dendrites, glomeruli comprise the processes of local interneurons and astrocytes, whose somata are surrounding the glomerular neuropil in which they project (Valverde and Lopez-Mascaraque, 1991; Berkowicz et al., 1994; Ennis et al., 1996; De Saint Jan and Westbrook, 2005; De Saint Jan et al., 2009; Lohr et al., 2014). In the glomeruli, the first level of neuronal processing of odor information is established by feed-forward excitation and feedback inhibition within the axo-dendritic and dendro-dendritic glomerular network. The somata of tufted cells lie in the lower part of the glomerular layer and the adjacent so-called external plexiform layer. Below the external plexiform layer, the cell bodies of mitral cells are located in a single-rowed layer, the mitral cell layer. Mitral cells possess a characteristic morphology with a relatively large, pyramidal cell body and an apical dendrite ending in a single glomerulus. They have few lateral dendrites, branching in the external plexiform layer, where they build reciprocal dendro-dendritic synapses with granule cells and other local interneurons. Thus, the external plexiform layer demarcates a second area of odor signal processing besides the glomerular layer, meaning that mitral/tufted cells integrate two spatially and functionally independent levels of information processing. Just below the mitral cell layer, the axons of mitral/tufted cells assemble and proceed caudally, forming the internal plexiform layer. The deepest layer of the olfactory bulb is mainly filled with granule cells, small axon-less interneurons that project their dendrites to the external plexiform layer.

### Primary Olfactory Cortex

On their way towards the cortical areas, the axons of mitral/tufted cells converge to the lateral olfactory tract, leaving the olfactory bulbs to project to areas of higher ordered sensory processing. The olfactory pathway shows some unique features compared to neuronal pathways of other senses such as vision, hearing and somatosensation. The main part of the information from the olfactory bulb is conveyed straight to cortical areas without passing a thalamic relay first (Gottfried, 2006). These cortical areas, receiving direct input from the olfactory bulb, are designated primary olfactory cortex and extend to the dorsomedial surface of the temporal lobe. As main elements, the primary olfactory cortex comprises (from rostral to caudal) the anterior olfactory nucleus (a.k.a. anterior olfactory cortex), olfactory tubercle, tenia tecta, piriform cortex, anterior cortical nucleus of the amygdala and entorhinal cortex (Figure 1). Apart from the entorhinal cortex, the areas of the primary olfactory cortex consist of a three-layered allo-(palaeo-)cortex, pointing to its ancient evolutionary origins (Gottfried, 2006). Notably, all these regions are not exclusively destined to process olfactory information, but, being elements of the limbic system, rather fulfil multiple functions such as processing emotions and memory formation, which can anecdotally be experienced as highly vivid and emotional autobiographical memories triggered by olfactory sensation (Gottfried, 2006; Wilson et al., 2014). The connection between primary olfactory cortex structures and the olfactory bulb is reciprocal, apart from the olfactory tubercle, a structure which is rudimentary in humans and does not feed back to the olfactory bulb (Gottfried, 2006). Most subregions of the primary olfactory cortex are intensely interconnected *via* cortical fibers with each other and with higher-ordered secondary cortical areas such as the orbitofrontal cortex, mediodorsal thalamus and amygdala (Wilson et al., 2014). A key area of this network producing olfactory perception is the piriform cortex. Despite the relatively simple three-layered cytoarchitecture, the piriform cortex executes diverse operations, which are attributed to higher-ordered associative cortices rather than to primary sensory cortices. For instance, the piriform cortex contributes to decorrelation of odor responses in order to discriminate different odors, pattern completion allowing perceptual stability and encoding the value associated with the odor creating an odor context memory (Haberly, 2001; Courtiol and Wilson, 2017).

## Sources and Breakdown of ATP and Adenosine

ATP, as intracellular energy supply, is present in all cells and therefore ubiquitously available as an intercellular signaling molecule. Thus, the presence of high amounts of ATP in the extracellular space as a result of injury or cell death seems to be a common signal of cell damage (Burnstock and Verkhratsky, 2009). Apart from that, a controlled release of ATP by exocytosis, active transport or membrane pores is also known for most brain areas (North and Verkhratsky, 2006; Lohr et al., 2011; Burnstock, 2013). Depending on the release pathway, achieved local concentration, the rate of degradation or uptake and the presence of different types of purinoceptors, ATP and other nucleotides transduce or modulate physiological responses in a highly specific manner.

In the olfactory system, the active release of ATP, ADP or adenosine has been described in the olfactory epithelium, the olfactory bulb and the olfactory cortex. ATP is released by exocytosis in the olfactory epithelium and activates both P2Y and P2X receptors (Hegg et al., 2009; Hayoz et al., 2012). In the olfactory bulb, electrical stimulation of axons of olfactory sensory neurons results in co-release of ATP and glutamate at the axon terminals in the glomerular layer as well as alongside the axons in the nerve layer, which was termed “ectopic” release (Thyssen et al., 2010). The concentration of extracellular ATP released from these neurons was estimated to reach the upper micromolar range (Thyssen et al., 2010). The release of ATP from olfactory sensory neurons depends on a rise in intracellular calcium and is suppressed by impairing exocytosis with botulinum toxin, indicating vesicular release (Thyssen et al., 2010). In addition, astrocytes provide a source for extracellular ATP in the olfactory bulb. Roux et al. (2015) demonstrated the significance of astrocytic connexin 43 hemichannels for purinergic neuron-glia communication and baseline ATP levels in the mouse olfactory bulb. ATP-mediated effects on mitral cells are blocked in mice deficient in connexin 43, a connexin mainly expressed by astrocytes, and by the connexin 43-specific inhibitory peptide Gap26. On the other hand, ATP release from astrocytes *via* the opening of connexin 43 hemichannels is reduced by tetrodotoxin (TTX) and hence depends on neuronal activity (Roux et al., 2015). The prominent role of astrocytic hemichannels in controlling extracellular ATP is further confirmed by the finding that Gap26 results in a reduction of ambient ATP levels by about 50% (Roux et al., 2015).

Purinergic signaling is more complex than most other neurotransmitter systems, since extracellular degradation of ATP is not the endpoint of its action, but activates other parts of the purinergic signaling system. A variety of extracellular enzymes, e.g., tissue non-specific alkaline phosphatases and ecto-nucleotidases, catalyze the successive degradation of ATP (Zimmermann, 2006). The activity of these enzymes and of adenosine deaminase is notably high in the olfactory bulb, compared to other brain regions, suggesting a rapid extracellular breakdown from ATP to adenosine and great significance of ATP dephosphorylation products as signaling molecules in the olfactory bulb (Geiger and Nagy, 1987; Schoen and Kreutzberg, 1995, 1997; Clemow and Brunjes, 1996; Langer et al., 2008). Interestingly, the activity of different nucleotide-degrading enzymes differs significantly between different layers of the olfactory bulb. Ecto 5′-nucleotidase (CD73), e.g., has a very high activity in the external and internal plexiform layers as well as in the granule cell layer, while tissue non-specific alkaline phosphatase is active in the glomerular, external and internal plexiform layers (Langer et al., 2008). Physiological experiments in acute olfactory bulb slices provide additional evidence for the importance of tissue non-specific alkaline phosphatase, since its inhibition suppresses the A_2A_ receptor-dependent calcium response in periglomerular astrocytes upon application of ATP, while inhibition of ecto-nucleotidases with ARL 67156 had no effect (Doengi et al., 2008). A study by Pani et al. (2014) provides data about baseline levels of ATP, ADP, AMP and adenosine in five brain regions, including the olfactory bulb, of five different strains of mice. Using high-performance liquid chromatography with electrochemical detection, they found significant variations of baseline purine levels between brain regions and mouse strains. In the olfactory bulb, total adenosine concentrations varied significantly from about 3,000 pg/mg wet weight in C57BL/6J mice to about 600,000 pg/mg wet weight in BALB/c mice. In C57BL/6J mice, both adenosine and ATP showed the lowest concentrations in the olfactory bulb compared to the other brain regions such as the cerebral cortex and hippocampus (Pani et al., 2014). Yet, whether these variations in total purine concentration also reflect diversity in extracellular purine levels and purinergic neuromodulation in different brain areas or mouse strains remains an open question.

**Figure 2:**
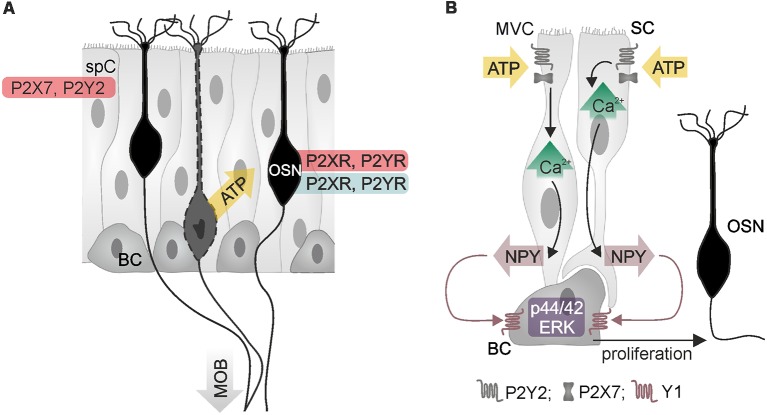
Purinergic signaling in the main olfactory epithelium. **(A)** Olfactory sensory neurons (OSN) and supporting cells (spC) express P2X and P2Y receptors, as demonstrated by physiological (red) and histological data (blue). Adenosine 5′-triphosphate (ATP) is released from injured or degenerating OSN (yellow arrow). BC, basal cell; MOB, main olfactory bulb. **(B)** ATP stimulates calcium signaling in two types of supporting cells, microvillar cells (MVC) and sustentacular cells (SC), resulting in the release of neuropeptide Y (NPY). Activation of Y1 NPY receptors of basal cells stimulates proliferation *via* p44/42 mitogen-activated protein kinase (extracellular signal-regulated kinase; ERK), which generates new OSN. Illustration by SciGraphics.

**Figure 3:**
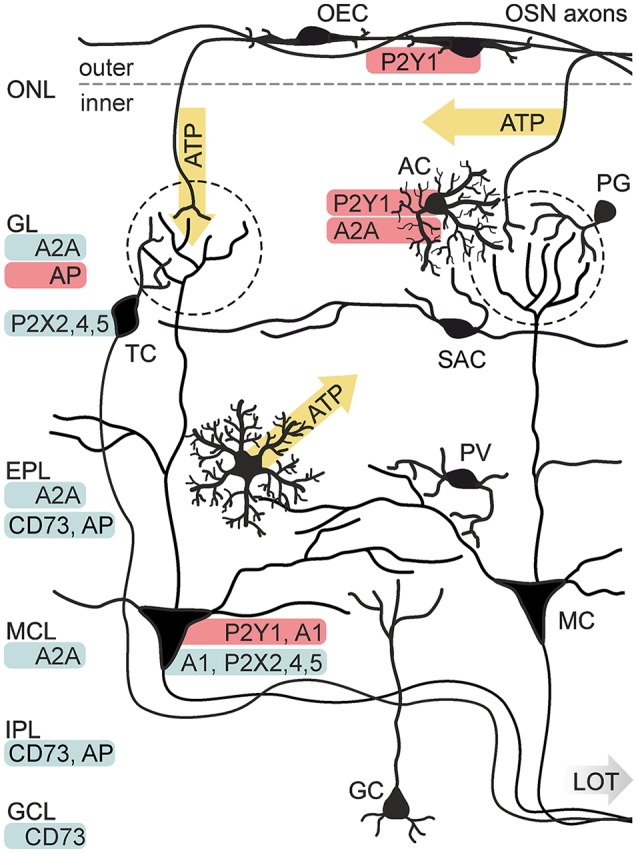
Purinergic signaling in the olfactory bulb. Known release sides of purines (yellow) and distribution (red: physiological data, blue: histological data) of purinoceptors and degrading enzymes in the cellular network of the olfactory bulb. Electrical stimulation of axons of olfactory sensory neurons (OSN) results in the release of ATP from axon terminals in the GL and alongside the axons in the ONL. ATP is released from astrocytes (AC) *via* the opening of connexin 43 hemichannels. P2X_2/4/5_ receptors have been identified histologically in mitral cells (MC) and tufted cells (TC). Physiological evidence exists for the expression of P2Y_1_ receptors in AC and MC, as well as for A_1_ receptor expression in MC and A_2A_ receptor expression in AC. Expression of the A_2A_ receptor has been shown histologically for the GL, EPL and mitral cell layer (MCL). Activity of nucleotide-degrading enzymes alkaline phosphatase (AP) and ecto 5′-nucleotidase (CD73) has been demonstrated in several layers. Abbr.: GC, granule cell; GCL, granule cell layer; IPL, internal plexiform layer; LOT, lateral olfactory tract; OEC, olfactory ensheathing cell; PG, periglomerular neuron; PV, parvalbumin-positive neuron; SAC, short axon cell. Illustration by SciGraphics.

## Purinergic Signaling in the Olfactory Epithelium

The olfactory epithelium mainly consists of sensory neurons, sustentacular cells, microvillar cells and basal cells (Figure 2). All four cell types have been found to express purinoceptors (Hegg et al., 2003). Immunohistochemical, electrophysiological and calcium imaging studies demonstrated the presence of P2X and P2Y receptors in olfactory sensory neurons (Hegg et al., 2003). Application of ATP excites olfactory sensory neurons and leads to an increase in the cytosolic calcium concentration in explants of the olfactory epithelium of mice and *Xenopus* tadpoles (Hegg et al., 2003; Czesnik et al., 2006). Despite the excitatory effect of ATP on non-stimulated olfactory sensory neurons, calcium responses upon stimulation by odors are inhibited by co-application of ATP (Hegg et al., 2003). In line with this, odor-evoked responses in olfactory sensory neurons are increased in amplitude when purinoceptors are blocked by the non-specific P2 antagonists PPADS and suramin, indicating purinergic modulation of odor responses by endogenously released ATP (Hegg et al., 2003).

Besides the impact on sensory signal transduction, purinergic agonists such as ATP, ADP and UTP also evoke calcium signaling in sustentacular cells and microvillar cells, linking neuronal activity and trophic functions (Hegg et al., 2003, 2009; Czesnik et al., 2006; Hassenklöver et al., 2008; Jia and Hegg, 2015). In sustentacular cells, ATP and UTP stimulate calcium signaling *via* P2Y receptor-mediated calcium release from internal stores (Hassenklöver et al., 2008; Hegg et al., 2009). These calcium signals occur as calcium oscillations in individual cells but can also travel as calcium waves through the gap junction-coupled syncytium of sustentacular cells in the olfactory epithelium (Vogalis et al., 2005a; Hassenklöver et al., 2008; Hegg et al., 2009). Calcium transients in sustentacular cells are accompanied by activation of calcium-dependent BK potassium channels, which results in hyperpolarization of the cell membrane (Vogalis et al., 2005a,b). Degeneration of sensory neurons in the olfactory epithelium evokes the release of ATP (Jia and Hegg, 2010; Hayoz et al., 2012). In neonatal mice, this ATP release is potentiated by ATP-induced ATP release, which involves P2X_7_ and P2Y receptors and results in extracellular ATP concentrations in the low micromolar range (Hayoz et al., 2012). Besides sustentacular cells, the second type of supporting cells, microvillar cells, resides in the olfactory epithelium and expresses purinoceptors (Hegg et al., 2010; Fu et al., 2018). Extracellular ATP evokes calcium signaling in both, sustentacular and microvillar cells, by activation of P2Y_2_ and P2X_7_ receptors (Figure 2). A subpopulation of microvillar cells that expresses type 3 inositol trisphosphate receptor (IP3R3) and transient receptor potential channel M5 (TRPM5) also expresses the neurotrophic factor neuropeptide Y (NPY); in these cells, P2 receptor-dependent calcium signaling leads to release of NPY (Montani et al., 2006; Kanekar et al., 2009; Jia and Hegg, 2015). Activation of Y1 NPY receptors in basal cells stimulates p44/42 extracellular signal-regulated kinase (ERK), thereby promoting proliferation of basal cells that differentiate into mature olfactory sensory neurons (Jia et al., 2009, 2013; Jia and Hegg, 2012). Upon maturation, these sensory neurons grow their axons into the olfactory bulb and form synapses with mitral and tufted cells.

## Purinergic Signaling in the Olfactory Bulb

### Purinoceptor Expression

With a growing number of studies showing the influence of purinergic signaling in olfaction, a widespread involvement of purinoceptors in this sensory system becomes obvious. By now, the presence of purinoceptors has been shown for all layers of the olfactory bulb by means of physiological studies, antibody staining, *in situ* hybridization and binding studies with radio-labeled receptor ligands (Figure 3). P2X_2_, P2X_4_, P2X_5_ and P2X_6_ receptor expression has been demonstrated in the principal neurons of the olfactory bulb, mitral and tufted cells, by *in situ* hybridization and immunolabeling, while P2X receptors have so far not been found in glial cells of the olfactory bulb (Collo et al., 1996; Vulchanova et al., 1996; Lê et al., 1998; Guo et al., 2008; Kaneda et al., 2010). For the family of P2Y receptors, only little information about expression in the olfactory bulb is published. Staining with a radio-labeled P2Y_1_ ligand showed positive results for all layers of the olfactory bulb, with a pronounced staining in the glomerular layer (Simon et al., 1997). In olfactory bulb tissue, mRNA expression of all four types of adenosine receptors has been demonstrated by Northern blot (Mahan et al., 1991; Dixon et al., 1996). A_1_ receptors, as well as A_2A_ receptors have been found by *in situ* hybridization and antibody staining (Johansson et al., 1997; Rosin et al., 1998; Kaelin-Lang et al., 1999). Noteworthy, the expression levels of A_1_ and A_2A_ were among the highest in the entire mouse brain. In the olfactory bulb mRNA of A_1_ receptors is predominantly expressed in a pattern resembling the localization, size and abundance of mitral and tufted cells (Rotermund et al., 2018). *In situ* hybridization depicts a more widespread expression of the A_2A_ receptor, with intense staining in mitral and tufted cells, but also in periglomerular interneurons and granule cells (Rotermund et al., 2018).

### Glial Cell Physiology and Function

Purinergic signaling in the olfactory bulb has first been described in glial cells, corroborating the important role of purines for neuron-glia interaction. Olfactory ensheathing cells, exclusively found in the olfactory nerve including the olfactory bulb nerve layer, respond to application of ATP with a P2Y_1_-mediated calcium increase in neonates (Rieger et al., 2007; Thyssen et al., 2010) as well as in adults (Thyssen et al., 2013; Stavermann et al., 2015). In contrast, application of adenosine has no impact on calcium levels in these cells (Thyssen et al., 2013). A recent study demonstrates that the observed effect of P2Y_1_-mediated calcium signaling is restricted to P75 neurotrophin receptor-positive olfactory ensheathing cells of the outer nerve layer, whereas P75-negative olfactory ensheathing cells of the inner nerve layer do not generate calcium signals in response to ATP or other purinergic ligands (Thyssen et al., 2013; Stavermann et al., 2015). Like most other neurotransmitter-mediated calcium responses in glial cells, the P2Y_1_-mediated intracellular calcium increase in olfactory ensheathing cells is based on calcium release driven by IP3 receptors, being amplified by calcium-induced calcium release and prolonged by store-operated calcium entry (Stavermann et al., 2012; Thyssen et al., 2013).

In addition to olfactory ensheathing cells, olfactory bulb astrocytes express purinoceptors. Periglomerular astrocytes have been shown to possess P2Y_1_ and A_2A_ receptors that are linked to intracellular calcium signaling (Doengi et al., 2008; Lohr and Deitmer, 2010). ATP, which is co-released with glutamate at axon terminals of olfactory sensory neurons, is degraded extracellularly to ADP and adenosine. It was shown that both ADP and adenosine elicit a rise in intracellular calcium levels in olfactory bulb astrocytes, mediated by P2Y_1_ and A_2A_ receptors, while the co-released glutamate activates metabotropic glutamate receptor 5 (mGluR_5_) and calcium-permeable AMPA receptors in these cells (Petzold et al., 2008; Verkhratsky et al., 2009; Droste et al., 2017). Calcium signaling evoked by mGluR_5_ activation is only found in astrocytes of developing animals, while the P2Y_1_-, A_2A_- and AMPA receptor-evoked calcium responses persist in adults (Doengi et al., 2008; Otsu et al., 2015; Droste et al., 2017; Beiersdorfer et al., 2019). In accordance with calcium signaling in olfactory ensheathing cells, ATP- and ADP-evoked rises in intracellular calcium in olfactory bulb astrocytes are driven by calcium release *via* IP3 signaling and subsequent calcium entry by the activation of store-operated calcium channels (Singaravelu et al., 2006; Doengi et al., 2009). Interestingly, the calculated EC50 (4.5 μM) for ADP-induced calcium signaling in glomerular astrocytes is higher by more than one order of magnitude compared to the EC50 value (0.23 μM) for A_2A_ receptor-dependent calcium signals (Doengi et al., 2008). This concentration-dependent activation may result in a differentiated activation of purinergic receptors: only high amounts of ATP above 1 μM are sufficient to activate P2Y_1_ receptors in glomerular astrocytes, whereas lower amounts of ATP bypass P2 receptor activation and directly target adenosinergic P1 receptors providing the possibility of graduated responses to different neuronal activity.

In other brain areas, astrocytes have been shown to respond to neurotransmitters released from presynaptic terminals with increases in the cytosolic calcium concentration, which can result in release of so-called gliotransmitters (e.g., glutamate, D-serin and ATP) and other substances such as arachidonic acid and prostaglandins which affect adjacent synapses and blood vessels (Haydon and Carmignoto, 2006; Attwell et al., 2010; Verkhratsky et al., 2012; Shigetomi et al., 2016; Dallérac et al., 2018). Calcium-dependent modulation of blood vessels by astrocytes upon neuronal activity is called neurovascular coupling and has also been studied in the olfactory bulb (Petzold et al., 2008; Doengi et al., 2009; Lohr et al., 2011; Otsu et al., 2015). Neurovascular coupling is an important mechanism to cope with the increasing energy demand upon neuronal activity, since action potential firing and synaptic transmission are the main energy sinks in the brain (Attwell and Laughlin, 2001; Attwell and Gibb, 2005; Barros and Deitmer, 2010). In addition, neurovascular coupling regulates and stabilizes the blood pressure in brain arterioles (Iadecola and Nedergaard, 2007). In olfactory bulbs of living, anesthetized mice, odor stimulation evokes mGluR_5_- and P2Y_1_/A_2A_-dependent calcium signaling in astrocytes that trigger dilation of adjacent capillaries and hyperemia (Petzold et al., 2008; Otsu et al., 2015). However, since mGluR_5_ is down-regulated in astrocytes of the cortex and the olfactory bulb in adult mice, glial mGluR_5_ is unlikely to mediate neurovascular coupling in adult animals (Sun et al., 2013; Otsu et al., 2015; Beiersdorfer et al., 2019). In contrast, calcium signaling evoked by ATP and possibly ADP and adenosine persists from neonates to adults (Doengi et al., 2008; Beiersdorfer et al., 2019), resulting in astrocytic calcium responses upon odor stimulation even in the absence of mGluR_5_ (Otsu et al., 2015). Calcium transients in astrocytic microdomains of perivascular endfeet are followed by a drop in calcium in perivascular pericytes and, finally, vasodilation as well as hyperemia in the glomerular layer (Otsu et al., 2015; Rungta et al., 2018). The olfactory nerve layer comprises axons of olfactory sensory neurons and olfactory ensheathing cells but lacks astrocytes with the exception of astrocytic processes that enwrap arterioles that penetrate the nerve layer. Calcium signaling in astrocytes around penetrating arterioles is able to mediate neurovascular coupling (Petzold et al., 2008), however, capillaries in the nerve layer require a different mechanism to adapt to neuronal activity. Capillaries in the nerve layer are enwrapped by the fine processes of olfactory ensheathing cells (Thyssen et al., 2010). Olfactory ensheathing cells express mGluR_1_ as well as P2Y_1_ receptors and respond to glutamate and ATP released from axons of sensory neurons with calcium transients (Rieger et al., 2007; Thyssen et al., 2010, 2013). Similar to astrocytes, mGluRs are down-regulated during the first postnatal weeks, whereas purinoceptor expression persists until adulthood (Thyssen et al., 2013; Stavermann et al., 2015; Beiersdorfer et al., 2019). Neuronal activity, local stimulation of P2Y_1_ receptors and induction of calcium signaling in single olfactory ensheathing cells trigger vasoresponses in the olfactory bulb capillaries, indicating that in addition to astrocytes, olfactory ensheathing cells are able to mediate neurovascular coupling *via* purinergic signaling (Thyssen et al., 2010; Lohr et al., 2011, 2014; Beiersdorfer et al., 2019).

**Figure 4:**
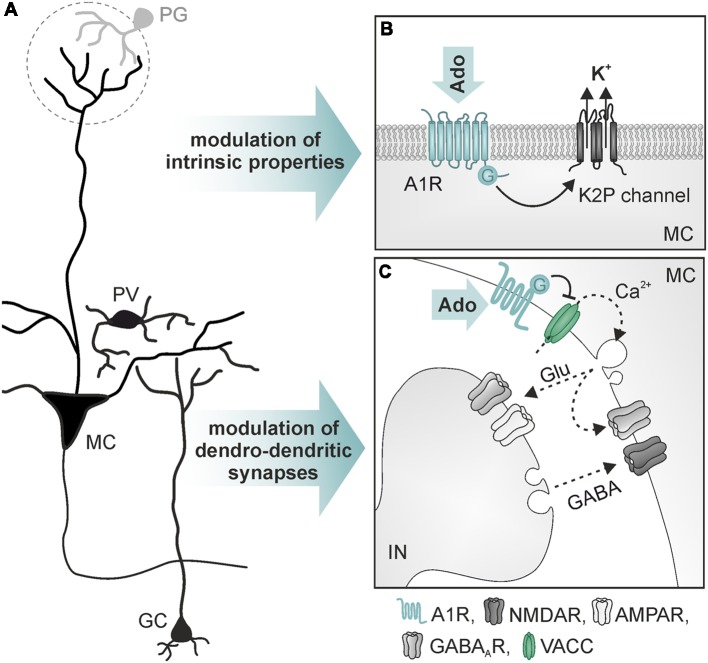
Mechanisms of action of adenosine in mitral cells. **(A)** Simplified neuronal network in the olfactory bulb. **(B)** Opening of two-pore domain potassium channels (K2P) by activation of A_1_ receptors leads to hyperpolarization of mitral cells (MC). **(C)** A_1_ receptor-mediated reduction of Ca^2+^ influx into presynaptic sites of MC reduces dendro-dendritic inhibition at reciprocal synapses between MC and interneurons (IN; GC and parvalbumin-positive interneurons). Abbr.: Ado, adenosine; Glu, glutamate; GC, granule cells; PG, periglomerular interneuron; PV, parvalbumin-positive interneuron; VACC, voltage-activated calcium channel. Illustration by SciGraphics.

### Neuromodulation

ATP and its breakdown products ADP and adenosine have been described as neuromodulators in many instances (Chen et al., 2014; Sebastião and Ribeiro, 2015; Guzman and Gerevich, 2016; Köles et al., 2016; Boué-Grabot and Pankratov, 2017). In developing as well as mature olfactory bulbs, ATP increases neuronal network activity leading to excitation of output neurons, the mitral and tufted cells (Fischer et al., 2012; Schulz, 2018). In these studies, wide-field photoapplication of ATP (by photolysis of caged ATP) evokes an increase of synaptic inputs and excitation of mitral cells, measured as whole-cell current and voltage responses. In addition, local release of ATP by photoapplication in a single glomerulus produces an increase of synaptic inputs in the mitral cell which is accompanied by an increase in intracellular calcium in the mitral cell tuft (Fischer et al., 2012). The ATP response depends on the activation of P2Y_1_ receptors, as inhibition of P2Y_1_ receptors by the specific antagonist MRS2179 impeded the effect. The rise in network activity is not the consequence of direct P2Y_1_-mediated excitation of the mitral cell itself, but of other neurons, since suppression of neuronal firing with TTX and inhibition of NMDA and AMPA receptors, respectively, not only abolished the network response but also eliminated P2Y_1_-mediated current responses in mitral cells almost entirely (Fischer et al., 2012). So far, the identity of these P2Y_1_-expressing neurons has not been resolved. Despite the high expression of P2X receptors in mitral and tufted cells, as shown by *in situ* hybridization and immunolabeling (Lê et al., 1998; Kanjhan et al., 1999; Guo et al., 2008), P2X-mediated currents could not be measured yet (Figure 3). In summary, the studies show that purinoceptors are abundant in the olfactory bulb and ATP has a strong excitatory effect on neuronal network activity, however, the exact mechanisms by which ATP modulates neuronal performance still need to be elucidated.

**Figure 5:**
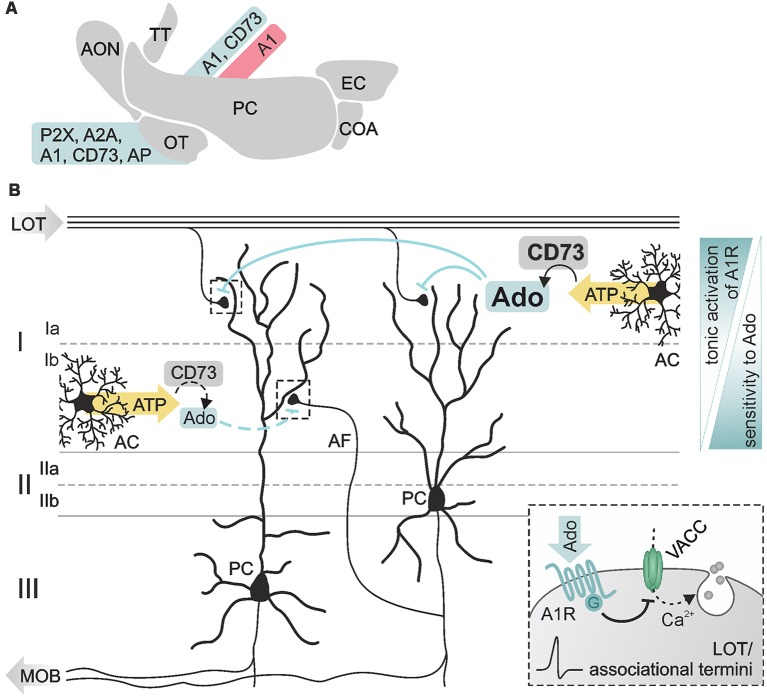
Purinergic modulation in the piriform cortex. **(A)** Expression of members of the purinergic signaling pathway in the olfactory cortex documented by physiological (red) and histological (blue) data. **(B)** Pyramidal cells (PC) reside in layer II/III of the piriform cortex with dendritic arbors spreading in superficial layer I. Synaptic inputs at PC dendrites are segregated in distinct sublayers, sublayer Ia exclusively receiving excitatory input from fibers of the LOT, sublayer Ib receiving input from intracortical associational fibers (AF). Adenosine affects both types of inputs by A1 receptor-dependent inhibition of presynaptic voltage-activated calcium channels (VACC) and thereby inhibition of glutamate release (see inset). Ecto 5′-nucleotidase (CD73) is differentially expressed in the sublayers resulting in higher basal levels of extracellular adenosine in layer Ia compared to layer Ib. Synaptic transmission in both layers shows different sensitivity to adenosine, associative synapses in layer Ib being more sensitive to adenosine than LOT synapses in layer Ia. Abbr.: AC, astrocyte; MOB, main olfactory bulb. Illustration by SciGraphics.

A well-established role of adenosine is the regulation of glutamatergic synaptic transmission by activation of presynaptic P1 receptors (Sebastião and Ribeiro, 2009; Chen et al., 2014). In the olfactory bulb, A_1_ and A_2A_ receptors are highly expressed (Kaelin-Lang et al., 1999; Rotermund et al., 2018). In addition, the activity of ATP-degrading ectoenzymes required for the production of adenosine from ATP is among the highest activities found in the rodent brain (Langer et al., 2008). It has been shown that adenosine acts as a neuromodulator in the olfactory bulb (Roux et al., 2015; Rotermund et al., 2018; Schulz et al., 2018; Figure 4). Whole cell patch clamp recordings show that activation of A_1_ receptors hyperpolarizes mitral cells and reduces the network activity in olfactory bulb brain slice preparations of wild type mice, but not of A_1_ receptor knockout mice (Rotermund et al., 2018). The hyperpolarization is mediated by increasing the potassium conductance of the mitral cell membrane. While in other brain areas, A_1_ receptor activation leads to stimulation of G protein-coupled inwardly rectifying potassium (GIRK) channels (Sickmann and Alzheimer, 2003; Kim and Johnston, 2015), the effect of adenosine on mitral cells is based on an A_1_ receptor-dependent opening of two-pore domain potassium channels (Figure 4B), probably reflecting very ancient cellular mechanisms in this archicortex (Rotermund et al., 2018). Roux et al. (2015) found constitutive release of ATP from astrocytes that is degraded to adenosine, resulting in DPCPX-sensitive alteration of slow membrane oscillations of mitral cells. In another study, DPCPX had no effect on steady-state excitability of mitral cells but inhibited adenosine-evoked hyperpolarization (Rotermund et al., 2018). The adenosine-evoked hyperpolarization is associated with a decrease in spontaneous action potential firing. In contrast, bursting activity of mitral cells upon synaptic input from sensory fibers is unaffected by adenosine, indicating lack of adenosine-mediated plasticity at synapses between sensory neurons and mitral cells (Rotermund et al., 2018). This results in a significant increase in the ratio of synaptically evoked firing over spontaneous firing and hence, in the signal-to-noise ratio of the output signaling of mitral cells (Rotermund et al., 2018).

Mitral and tufted cells establish numerous reciprocal dendro-dendritic synapses between their lateral dendrites and inhibitory interneurons (Figure 4C), mainly granule cells and parvalbumin-positive cells (Crespo et al., 2013). Mitral cells express A_1_ receptors that are located in the presynaptic membrane of the mitral-to-interneuron synaptic connection of these reciprocal synapses. Activation of A_1_ receptors inhibits presynaptic voltage-dependent calcium channels of the N- and P/Q-type, thus reducing glutamate release from mitral cell lateral dendrites (Schulz et al., 2018). Consequently, interneurons receive less excitation and thus respond with diminished GABA release, resulting in attenuated recurrent inhibition in mitral cells. The attenuation of NMDA, as well as AMPA receptor-mediated dendro-dendritic inhibition, indicates that reciprocal synapses of mitral cells with both, granule cells (NMDA receptor-driven) and with parvalbumin-positive cells (AMPA receptor-driven), are modulated by adenosine (Schulz et al., 2018). Recurrent inhibition between mitral cells and GABAergic interneurons is fundamental for odor discrimination. Increasing recurrent inhibition by, for instance, optogenetic excitation of granule cells or enhanced GABA release from granule cells due to ablation of GluA2 subunits (thereby amplifying glutamate-evoked calcium influx) improves odor discrimination, whereas inhibition of granule cells impairs odor discrimination (Abraham et al., 2010; Gschwend et al., 2015). Consequently, A_1_ receptor-deficient mice that lack adenosine-mediated depression of recurrent inhibition outperform wild type mice in a behavioral olfaction test, emphasizing the role of adenosine in olfactory information processing (Schulz et al., 2018).

## Purinergic Signaling in the Olfactory Cortex

### Expression of Purinoceptors and Nucleotidases

As already known for many years, adenosine modulates synaptic transmission in the olfactory cortex (Kuroda, 1978; Scholfield, 1978; Okada and Kuroda, 1980; Collins and Anson, 1985) There is a number of studies demonstrating the expression of enzymes and receptors of the purinergic signaling cascade in the primary olfactory cortex (Figure 5A). Kidd et al. (1995) detected P2X receptor mRNA in the olfactory tubercle, implying a contribution of ATP to fast neurotransmission in this brain area. The majority of available data, though, concerns adenosinergic signaling. In the olfactory tubercle, the expression of adenosine A_2A_ receptors is the highest apart from expression in the striatum and the olfactory bulb as shown by *in situ* hybridization, antibody staining and autoradiographic labeling studies (Johansson and Fredholm, 1995; Luthin et al., 1995; Rosin et al., 1998, 2003; DeMet and Chicz-DeMet, 2002; Rotermund et al., 2018). A_1_ receptors are also expressed in the olfactory tubercle, albeit to a lesser degree (Fastbom et al., 1987; Rotermund et al., 2018). Antibody staining of the ecto-nucleotidase CD73 clearly labels the olfactory tubercle (Kulesskaya et al., 2013). Furthermore, enzymatic activity of ecto 5′-nucleotidases and alkaline phosphatase is very high in the olfactory tubercle, emphasizing the role of purinergic neurotransmission and -modulation in this brain area (Langer et al., 2008).

### Modulation of Synaptic Plasticity

Expression of A_1_ receptors and ecto 5′-nucleotidases has been found in the piriform cortex, pointing to a role of adenosine in neuromodulation (Goodman and Synder, 1982; Trieu et al., 2015). The main targets of adenosinergic modulation in the piriform cortex are excitatory synaptic inputs in pyramidal cell dendrites (Rezvani et al., 2007; Yang et al., 2007; Trieu et al., 2015; Perrier et al., 2019). The somata of pyramidal cells reside in layer II/III of the piriform cortex, while their apical dendritic arbors extend into the superficial layer I (Figure 5B). There they receive spatially segregated excitatory input from different sources: axons of mitral cells carry odor information *via* the lateral olfactory tract and synapse on pyramidal cell dendrites in layer Ia (Figure 5B, LOT), while intrinsic afferent fibers from piriform cortex pyramidal cells and other cortical areas (forming the piriform cortex association network) establish synapses with pyramidal cell dendrites in layer Ib (Figure 5B, AF; Luskin and Price, 1983a,b; Nevill and Haberly, 2004). Stimulation of lateral olfactory tract fibers evokes field excitatory postsynaptic potentials (fEPSP) in the piriform cortex with a characteristic bipolar waveform reflecting the two different kinds of input in pyramidal cells (Haberly, 1973). The initial peak represents the monosynaptic sensory transmission (lateral olfactory tract-to-pyramidal cell), followed by a compound signal representing the activity of associational network fibers within the piriform cortex (pyramidal cell-to-pyramidal cell). Both types of synapses are modulated by adenosine; in *in vivo* experiments, activation of A_1_ receptors has been shown to reduce both the initial peak as well as the subsequent compound signal of the fEPSP (Rezvani et al., 2007). This depression of synaptic transmission is mediated by A_1_ receptor-dependent inhibition of presynaptic voltage-gated calcium entry *via* N- and P/Q-type calcium channels (Yang et al., 2007). In addition, adenosine increases paired-pulse facilitation in layer Ia while decreasing paired-pulse depression in layer Ib (Yang et al., 2007). Hence, A_1_ receptor-mediated inhibition of calcium channels decreases the release probability at these synapses. Interestingly, synapses between associational network fibers and pyramidal cells in layer Ib are more sensitive for modulation by experimentally added adenosine compared to synapses between lateral olfactory tract fibers and pyramidal cells in layer Ia, indicating a lower concentration of endogenous adenosine in layer Ib (Yang et al., 2007). The low ambient adenosine concentration at layer Ib synapses results from slow ATP-to-adenosine conversion due to low abundance of ecto 5′-nucleotidase and lack of synaptic ensheathment by glial cell processes, which provide one major source of ATP in the cortex (Haberly and Behan, 1983; Nevill and Haberly, 2004; Pankratov and Lalo, 2015; Trieu et al., 2015; Yi et al., 2017). Synapses between lateral olfactory tract fibers and pyramidal cell dendrites in layer Ia are not only depressed by adenosine, but can also be facilitated dependent on the frequency of synaptic activation (Perrier et al., 2019). While adenosine has no effect on the amplitude of fEPSP evoked by a train of stimuli at frequencies between 3 and 25 Hz, it strongly facilitates fEPSP at frequencies between 50 and 100 Hz (Perrier et al., 2019). Since the entire range of stimulation frequencies used in that study reflects frequencies naturally occurring during odor perception depending on, e.g., odor concentrations, adenosine might fine-tune odor perception in an intensity-dependent manner. However, while the studies above clearly demonstrate neuromodulatory functions of adenosine in synaptic transmission and plasticity in the olfactory cortex, its role in olfactory learning and memory formation remains to be shown.

## Conclusion

Purinergic signaling is a universal and versatile system of neurotransmission and -modulation. The extraordinary high expression of purinoceptors and nucleotide-degrading enzymes in regions of the olfactory pathway such as the olfactory bulb and olfactory tubercle emphasize the significance of purinergic signaling for this sensory system. P2X, P2Y and adenosine receptors are involved in adult neurogenesis in the olfactory epithelium, link neuronal activity to vascular responses in the olfactory bulb and modulate synaptic transmission in the olfactory bulb as well as the olfactory cortex. However, research on the functional role of purinergic signaling in olfaction has only started recently. Based on the recent physiological studies that reveal a broad range of specific purinoceptor-dependent effects, further research faces the challenge to link these findings to olfactory perception and behavior. In addition, given the fact that olfactory impairment is an early symptom in many neurodegenerative and neuroinflammatory diseases, the involvement of purinergic signaling in neuronal degeneration in the olfactory system is expected to move into focus in the future. The use of cell-specific knockout animals in combination with disease models and behavioral tests might be a promising strategy to gain insights into the mechanisms of purinergic neuromodulation in health and disease.

## Author Contributions

NR, DH and CL wrote the text. KS designed the figures. All authors edited the manuscript.

## Conflict of Interest Statement

The authors declare that the research was conducted in the absence of any commercial or financial relationships that could be construed as a potential conflict of interest.
